# Gene Mutations Associated With Clinical Characteristics in the Tumors of Patients With Breast Cancer

**DOI:** 10.3389/fonc.2022.778511

**Published:** 2022-04-14

**Authors:** Chunfang Hao, Chen Wang, Ning Lu, Weipeng Zhao, Shufen Li, Li Zhang, Wenjing Meng, Shuling Wang, Zhongsheng Tong, Yanwu Zeng, Leilei Lu

**Affiliations:** ^1^ Department of Breast Oncology, Tianjin Medical University Cancer Institute and Hospital, Tianjin, China; ^2^ Operations Department, Shanghai OrigiMed Co., Ltd., Shanghai, China

**Keywords:** estrogen receptor, progesterone receptors, human epidermal growth factor receptor 2, genomic mutations, a family history of cancer

## Abstract

**Background:**

Clinical characteristics including estrogen receptor (ER), progesterone receptor (PR), and human epidermal growth factor 2 (HER2) are important biomarkers in the treatment of breast cancer, but how genomic mutations affect their status is rarely studied. This study aimed at finding genomic mutations associated with these clinical characteristics.

**Methods:**

There were 160 patients with breast cancer enrolled in this study. Samples from those patients were used for next-generation sequencing, targeting a panel of 624 pan-cancer genes. Short nucleotide mutations, copy number variations, and gene fusions were identified for each sample. Fisher’s exact test compared each pair of genes. A similarity score was constructed with the resulting *P*-values. Genes were clustered with the similarity scores. The identified gene clusters were compared to the status of clinical characteristics including ER, PR, HER2, and a family history of cancer (FH) in terms of the mutations in patients.

**Results:**

Gene-by-gene analysis found that *CCND1* mutations were positively correlated with ER status while *ERBB2* and *CDK12* mutations were positively correlated with HER2 status. Mutation-based clustering identified four gene clusters. Gene cluster 1 (*ADGRA2*, *ZNF703*, *FGFR1*, *KAT6A*, and *POLB*) was significantly associated with PR status; gene cluster 2 (*COL1A1*, *AXIN2*, *ZNF217*, *GNAS*, and *BRIP1*) and gene cluster 3 (*FGF3*, *FGF4*, *FGF19*, and *CCND1*) were significantly associated with ER status; gene cluster 2 was also negatively associated with a family history of cancer; and gene cluster 4 was significantly negatively associated with age. Patients were classified into four corresponding groups. Patient groups 1, 2, 3, and 4 had 24.1%, 36.5%, 38.7%, and 41.3% of patients with an FDA-recognized biomarker predictive of response to an FDA-approved drug, respectively.

**Conclusion:**

This study identified genomic mutations positively associated with ER and PR status. These findings not only revealed candidate genes in ER and PR status maintenance but also provided potential treatment targets for patients with endocrine therapy resistance.

## Introduction

Breast cancer is the most common cancer among women. The age-standardized incidence of breast cancer even surpassed lung cancer worldwide (1, 2). Although the treatment of breast cancer has achieved great success, there are still 18%–46% of patients whose cancer would eventually develop into late-stage breast cancer (3, 4).

For patients with late-stage breast cancer or patients with unresectable tumors, traditional endocrine therapy and chemotherapy are used concerning the status of several important biomarkers including estrogen receptor (ER), progesterone receptor (PR), and human epidermal growth factor receptor 2 (HER2). With the development of precision medicine, genomic mutations are utilized as additional biomarkers in endocrine therapy and chemotherapy. For hormone-positive patients, inactivating NF1 mutations could be used as the prognostic biomarker of endocrine therapy resistance (5); for patients with triple-negative breast cancer, germline BRCA1/2 mutations could be employed to choose the appropriate medicine. For patients with triple-negative breast cancer with germline *BRCA1/2* mutations, carboplatin chemotherapy brought about more clinical benefits than standard docetaxel chemotherapy (6). The objective response rate of carboplatin chemotherapy was two times higher than that of docetaxel chemotherapy.

The above phenomenon drove us to rethink the background mechanism of different ER, PR, and HER2 statuses. As we know, genomic mutations play a significant role in cancer development. They could also take part in the maintenance of ER, PR, and HER2 status. Evidence shows that breast cancer patients with amplification of *CCND1* tended to be ER-positive and associated with worse 15-year survival (7, 8). Patients with *CCND1* amplification would need long-term treatment. Identifying genomic mutations associated with ER, PR, and HER2 status could help to develop new biomarkers and treatment. In this work, we enrolled 160 Chinese patients at identifying somatic mutations in a Chinese population associated with a family history of cancer (FH).

In this work, we enrolled 160 patients with breast cancer. Samples from the 160 patients were used for next-generation sequencing. Short nucleotide mutations, copy number variations, and gene fusions were identified for each sample. The association between gene mutation clusters and the status of ER, PR, and HER2 were studied. Further, the enrichment of genomic mutations in the Kyoto Encyclopedia of Genes and Genomes (KEGG) pathway and actionability for each patient group were inferred.

## Methods

### Patients and Samples

This study was approved by the Ethics Committee of Tianjin Medical University Cancer Institute and Hospital (ID: bc2021063). Patients included in this study were those with breast cancer, with written informed consent, and with successful sequencing results. There are 160 Chinese patients included. Patients were staged according to the TNM system. Samples from these patients were obtained from surgery and fixed in 4% neutral-buffered formalin at 4°C for 24 h. The processed samples were embedded in paraffin wax for storage.

### Immunohistochemistry and Fluorescence *In Situ* Hybridization

PR, ER, and HER2 expressions were first detected with immunohistochemistry (IHC). Briefly, a 3–5-μm slice of the wax sample was deparaffinized on the glass slide in a 60°C oven for 1 h and washed with xylene for 15 min. The sample was rehydrated with gradient concentrations (100%, 95%, 85%, and 75%) of alcohol. Four minutes of pressure cooking and 10 min of cold-water bath were used to induce antigen retrieval. Hydrogen peroxide (3%) was used to quench endogenous peroxidase activity for 30 min. The slides were blocked with goat serum (10%) for 30 min and stained with rabbit antibodies for ER, PR, and HER2. A secondary antibody biotinylated with horseradish peroxidase (HRP) was further stained for 25 min (K8002 kit, Dako, Santa Clara, CA). The slides were processed with diaminobenzidine for 5 min (K8002 kit, Dako, Santa Clara, CA). After washing with deionized water, hematoxylin was used to restain the slides. ER- and PR-positive rates of >1% were defined as ER+ and PR+, respectively. HER2 score = 3+ and HER2 score = 0/1+ were defined as HER2+ and HER2-, respectively. HER2 score = 2+ was further verified by fluorescence *in situ* hybridization (FISH). It was performed according to the manual of the PathVysion HER-2 DNA Probe Kit (Abbott Molecular Inc., IL, USA). The HER2 FISH result was interpreted according to the American Society of Clinical Oncology (ASCO)/College of American Pathologists (CAP) guidelines (9).

The tumors were classified into three hormonal subtypes according to ER, PR, and HER2 status. The three hormonal subtypes are the luminal subtype (ER+ or PR+), HER2-enriched subtype (ER- and PR-, HER2+), and TNBC subtype (ER-, PR-, and HER2-).

### Library Construction, Sequencing, and Mutation Calling

The library construction and sequencing were performed in the Clinical Laboratory Improvement Amendments (CLIA)/CAP-compliant Molecular Diagnostics Service Laboratory of Shanghai OrigiMed Co., Ltd. Methods were similar to our previous work (10, 11). Briefly, the formalin-fixed paraffin-embedded (FFPE) tissues were deparaffinized by heating in a 60°C oven for 1 h. As a control, white blood cells from the paired whole blood samples were separated by centrifugation.

DNA was extracted using KAPA (Kapa Biosystems, Wilmington, MA, USA) HyperPrep Kit (Illumina, San Diego, CA, USA). Samples with at least 50 ng of double-stranded DNA were applied in further library construction for both tumor tissues and paired white blood cells. A panel of 624 pan-cancer genes was targeted for amplification. To reduce errors from amplification, molecular identifiers (MIDs) were added to DNA segment ends. Barcodes were also added for multiplex sequencing. The prepared DNA libraries were sequenced on an Illumina NovaSeq 6000 sequencer (Illumina, San Diego, CA). Around both ends of the reads, 151 base pairs were read. The average sequencing depth was about 1,000×.

Raw reads were first trimmed for adaptors by Cutadapt (version 1.18). Reads were de-duplicated according to MID labels. The software Burrows–Wheeler alignment with maximal exact matches (BWA-MEM, version 0.7.9a) (12) was used to map all the reads onto University of California, Santa Cruz (UCSC) hg19 reference sequences. The alignment was further recalibrated by BaseRecalibrator of GATK (version 3.8). Short nucleotide mutations were called with Mutect2 (13) and varscan (14). Germline mutations were defined as those in the paired white blood cells but not in the known single-nucleotide polymorphism database (ESP6500, 1000 Genomes, gnomAD, and ExAC). Somatic mutations were retrieved by filtering out possible germline mutations. Germline mutations were obtained from each patient’s white blood sequencing and the single-nucleotide polymorphism databases. Further, copy number variations were called with CNVkit (15), and gene fusions were identified with an in-house pipeline (16).

Tumor mutational burden (TMB) was defined as the number of mutations per million effective coverage length of the genome (1.795717 million bases).

### Significantly Mutated Genes

Mutations could be preferentially present in specific genes because of gene length, genomic regions, and other patient characteristics. To reduce mutation bias, MutSigCV (17) was used to identify the significantly mutated genes. The calculation was performed using the service on the GenePattern website (18). Default parameters were used. The resulting *P*-values were adjusted by the Benjamin and Hochberg method for the correction of multiple testing errors.

### Actionable Annotation of Mutations

A mutation actionable database, OncoKB (19), was used to query the available drugs and their evidence level for each mutation. A python script released by OncoKB, MafAnnotator.py, was used to automate the query. The disease type was set to “BRCA.” According to the evidence level of confidence, actionable mutations were classified into six, namely, “Level_1”, “Level_2A”, “Level_2B”, “Level_3A”, “Level_3B”, and “Level_4”. Level_1 is the treatment with an FDA-recognized biomarker predictive of response to an FDA-approved drug; Level_2A is the treatment with standard-care biomarker predictive of response to an FDA-approved drug. Level_ 2B is the treatment with standard-care biomarker predictive of response to an FDA-approved drug in another indication but not the standard care in this indication. Level_3A is the treatment with compelling clinical evidence that supports the biomarker as being predictive of response to a drug in this indication. Level_3B is the treatment with compelling clinical evidence that supports the biomarker as being predictive of response to a drug in another indication. Level_4 is the treatment with compelling biological evidence that supports the biomarker as being predictive of response to a drug.

### Public Datasets

A muskoskeletal memorial sloan kettering (MSK) cohort (20) was also used to contrast the results in this study. This cohort included 1,756 patients with breast cancer. Data were downloaded from the cBioPortal (www.cbioportal.org).

### Statistical Analysis

Comparisons between categorical variables were performed using Fisher’s exact test. Continuous variables were compared by the Mann–Whitney U test. Multiple-testing comparisons were corrected with the Benjamin and Hochberg method. *P*-values below 0.05 were considered statistically significant. The similarity score of two factors was obtained by transforming *P*-values to “-log10(*P*-value)”. When analyzing the association between clinical characteristics and genomic mutations, the cases were omitted if missing records in the current analysis.

## Results

### Clinical Characteristics of the Patients

Between 2016 and 2018, 160 patients diagnosed with breast cancer were enrolled in this study (**Table 1**). The patients were about 50 years old, and 32.5% of them were at late-stage. About 50% of patients were either ER-positive or PR-positive and 28.8% of them were HER2-positive. Samples were harvested from 95 primary tumors (59.4%) and 63 metastatic tumors (39.4%). A family history of cancer (FH) was present in 31.3% of patients. The distribution of FH is shown in **Supplementary Figure 1A**. Lung cancer, liver cancer, breast cancer, endometrial cancer, and colorectal cancer were diagnosed mostly in family members of patients with breast cancer (**Supplementary Figure 1B**), which accounted for 34.2% of patients with FH.

**Table 1:** Clinical characteristics of patients.

	Overall (N=160)
**Age**	
Mean (SD)	49.4 (11.4)
Median [min, max]	50.0 [20.0, 79.0]
Missing	24 (15.0%)
**Stage**	
I	8 (5%)
II	25 (15.6%)
III	19 (11.9%)
IV	51 (31.9%)
Missing	57 (35.6%)
**ER**	
Negative	71 (44.4%)
Positive	73 (45.6%)
Missing	16 (10.0%)
**PR**	
Negative	70 (43.8%)
Positive	73 (45.6%)
Missing	17 (10.6%)
**HER2**	
Negative	90 (56.2%)
Positive	42 (26.2%)
Missing	28 (17.5%)
**Subtype**	
Luminal	87 (54.4%)
HER2	10 (6.3%)
TNBC	35 (21.9%)
Missing	28 (17.5%)
**Location**	
Primary	95 (59.4%)
Metastatic	63 (39.4%)
Missing	2 (1.2%)
**Family History**	
No	99 (61.9%)
Yes	50 (31.2%)
Missing	11 (6.9%)

Patients are classified into three subtypes according to ER, PR, and HER2 expression statuses: luminal (ER^+^ or PR^+^), HER2 (ER^-^, PR^-^, HER^+^), and TNBC (triple-negative breast cancer, ER^-^PR^-^HER2^-^).

### Mutation Landscape of Breast Cancer

**Figure 1:**
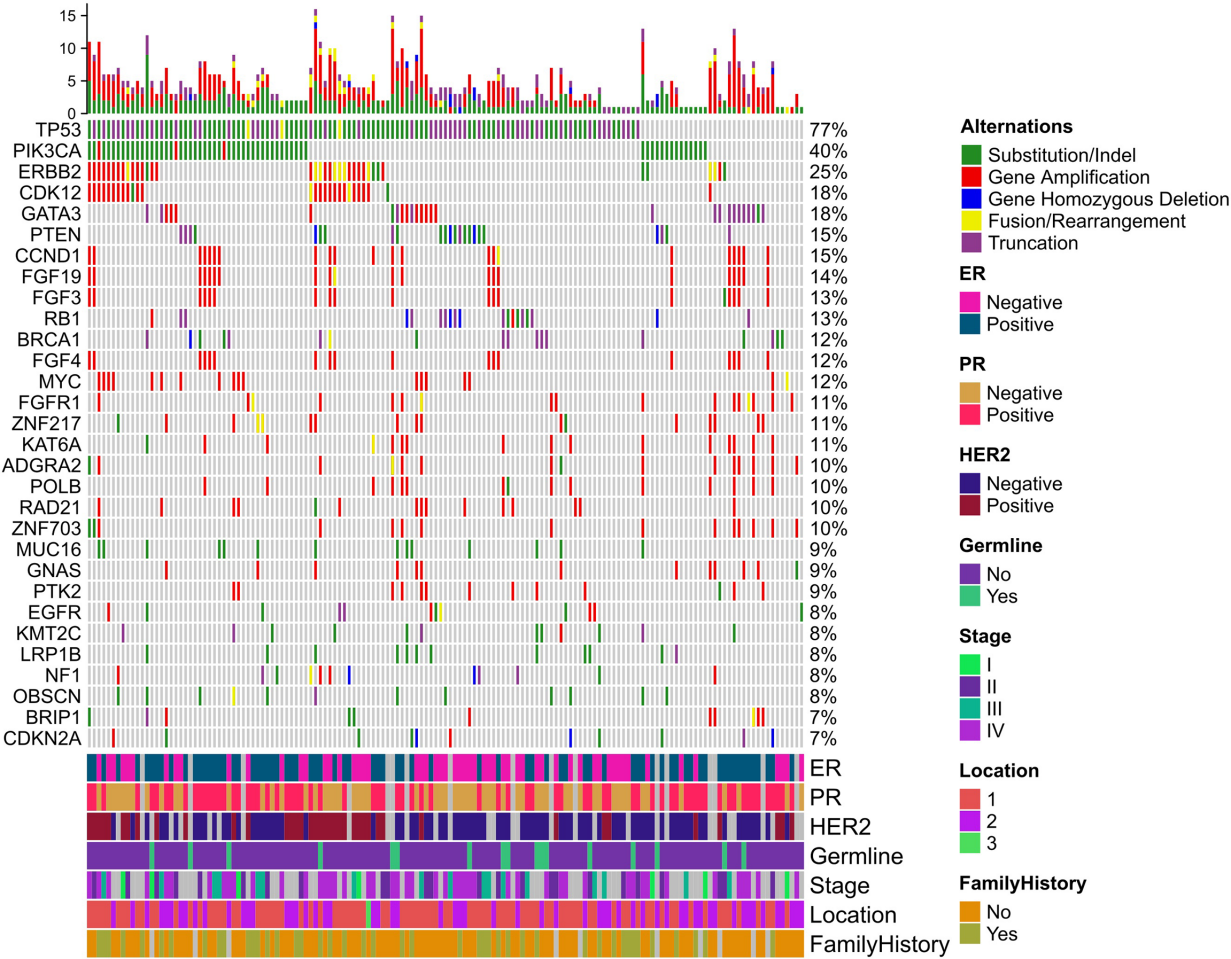
Mutational landscape of breast cancer. Five types of mutations are indicated with different colors in the heatmap plot. The top bar plot summarizes the mutation type proportion in each patient. The below color bar indicates the clinical characteristics of each patient. The heatmap shows the mutations at each gene in each patient.

**Figure 2:**
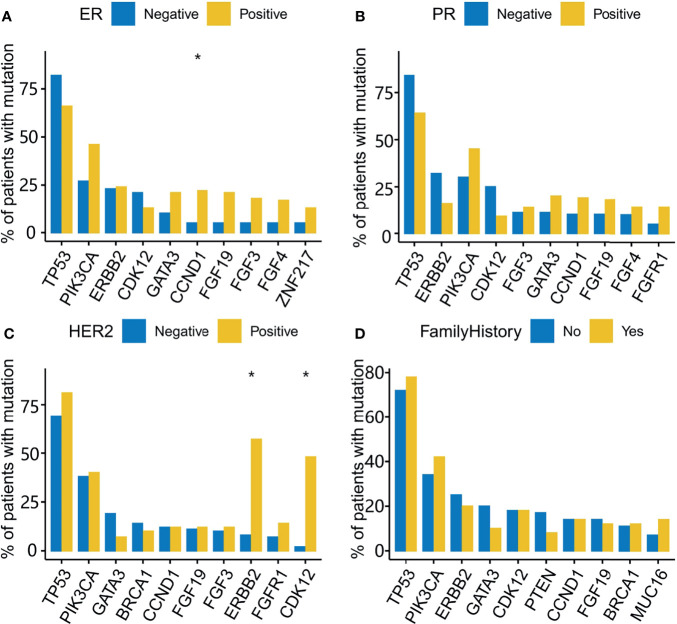
Gene mutations associated with ER, PR, HER2, and a family history of cancer (FH). The top 10 mutated genes are used in the analysis of mutational association with ER **(A)**, PR **(B)**, HER2 **(C)**, and FH **(D)**. The Y-axis indicates the percentage of patients with the mutated genes across the X-axis for receptor-positive and -negative groups. *, adjusted P-value<0.05.

he median percentage of effective coverage (depth >100) is 99.63% (standard error = 1.56), and the lowest is 90.3%. Short nucleotide genomic mutations in the tumors were called with Mutec2 (**Figure 1**). Mutations with allele frequency below 0.1% were filtered out for accuracy. The top-10 mutated genes were *TP53*, *PIK3CA*, *ERBB2*, *CDK12*, *PTEN*, *CCND1*, *FGF19*, *FGF3*, *RB1*, and *BRCA1* (**Figure 2**). Only six patients had germline mutations in genes including *BRCA1*, *BRCA2*, *ATM*, and *RAD51D*. For the top-mutated genes, the association between mutations and clinical characteristics including ER, PR, HER2, and FH is displayed in **Figures 2A–D**, respectively. *CCND1* was found significantly associated with ER status (**Figure 2A**) while *ERBB2* and *CDK12* were with HER2 status (**Figure 2C**). No significantly associated mutations were found significantly associated with PR (**Figure 2B**) and FH (**Figure 2D**). The association between gene mutations and breast cancer subtypes is shown in **Supplementary Figures 2A–C**. The HER-enriched subtype had a higher proportion of ERBB2 and CDK12 (**Supplementary Figure 2B**), and the TNBC subtype had a higher proportion of Kirsten Rat Sarcoma Viral Proto-Oncogene (KRAS) and a lower proportion of ERBB2 (**Supplementary Figure 2C**).

The results of this study were contrasted with those of the MSK cohort (20). In the MSK cohort, ER+ and PR+ patients had a higher proportion of *PIK3CA*, *CDH1*, *MAP3K1*, *AKT1*, *ESR1*, and *GATA3* mutations and a lower proportion of *TP53* mutations (**Supplementary Figures 2D, E**); HER2+ patients had a higher proportion of *TP53*, *NF1*, and *MLL2* and a lower proportion of *CDH1*, *GATA3*, and *MAP3K1* (**Supplementary Figure 2F**). The luminal subtype had a higher proportion of *PIK3CA*, *CDH1*, *GATA3*, and *MAP3K1* mutations and a lower proportion of *TP53* mutations (**Supplementary Figure 2G**), and the HER2-enriched subtype had a higher proportion of *TP53* and *NF1* mutations and a lower proportion of GATA3 mutations (**Supplementary Figure 2H**). No significant mutations were found in the TNBC subtype (**Supplementary Figure 2I**).

To reduce mutational bias from gene length, genomic region, and patient characteristics, etc., MutSigCV was used to identify the significantly mutated genes. Five genes (*TP53*, *PIK3CA*, *AKT1*, *PTEN*, and *GATA3*) were found significantly mutated with an adjusted *P*-value less than 0.05. None of these genes was significantly associated with ER, PR, HER2, and FH.

### Clustering Mutations

**Figure 3:**
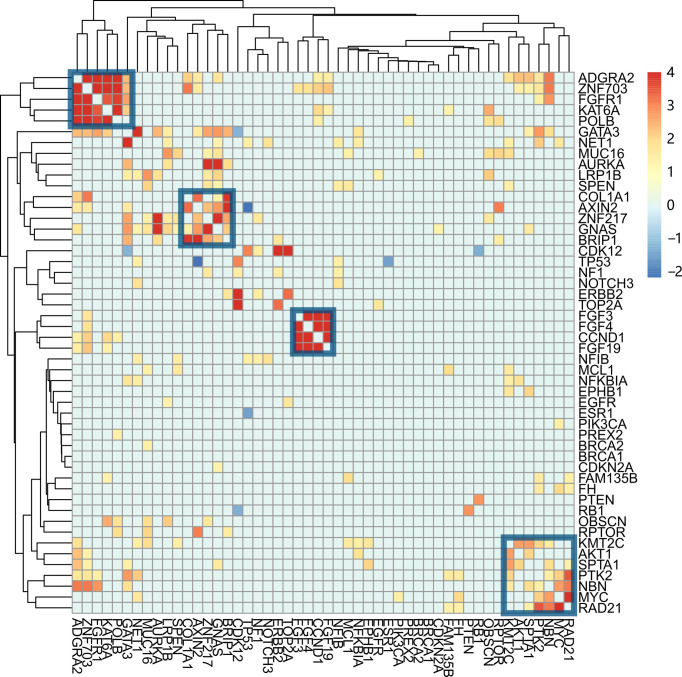
Co-mutation between genes and their association with clinical characteristics. The co-mutation association between each pair of mutations is calculated with Fisher’s exact test. The resulting *P*-value was transformed into a similarity score, -log10(*P*-value). Genes were hierarchically clustered with Euclidean distance and complete linkage. The four gene clusters are indicated with blue squares. From left to right, they are named clusters 1, 2, 3, and 4.

**Figure 4:**
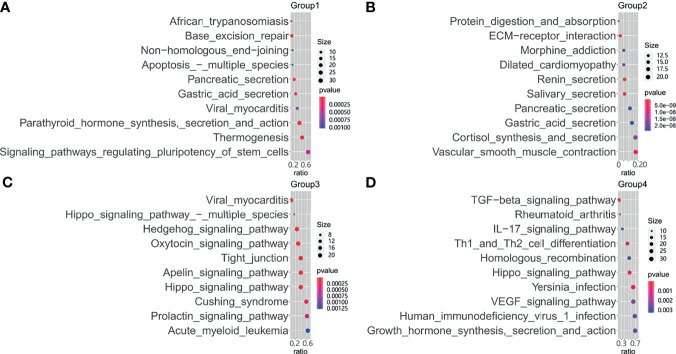
Functional enrichment of mutational genes. Enriched Kyoto Encyclopedia of Genes and Genomes (KEGG) pathways for mutated genes are plotted against the odds ratio in patient groups 1 **(A)**, group 2 **(B)**, group 3 **(C)**, and group 4 **(D)**.

Testing each gene's mutation frequency by Fisher's exact test had a low power to find out significant genes because of multiple testing problem. To improve statistical power, a clustering method was employed in advance. The clustering procedure included three steps. First, the association between two mutations was calculated with Fisher’s exact test. Second, the resulting *P*-values were transformed into a similarity score between two genes as “-log10(*P-value*)”. Third, the similarity scores were used to hierarchically cluster genes with Euclidean distance and complete linkage (**Figure 3**). From the heatmap result, four clusters having a high association within them were marked with rectangles. Patients were classified into four corresponding groups if there is a gene mutation in each gene cluster. Mutations within each group of patients were annotated with KEGG pathways. A hypergeometrical test was used to infer the significance of enrichment (**Figure 4**). Interestingly, group 1 was enriched with signaling pathways regulating pluripotency of stem cells (21) (**Figure 4A**); group 2 was enriched with protein digestion and absorption pathway and extracellular matrix (ECM)–receptor interaction pathway (22) (**Figure 4B**); group 3 was enriched with hippo signaling pathway (23) (**Figure 4C**); and group 4 was enriched with VEGF signaling pathway (24) (**Figure 4D**).

### Association of Mutational Clusters With Clinical Characteristics

**Figure 5:**
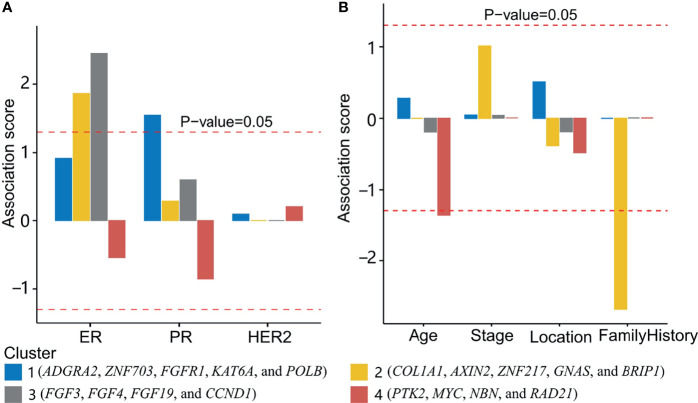
Association between gene clusters and clinical characteristics. **(A)** The association between gene clusters and three biomarkers (ER, PR, and HER2 status). Above the upper and below the lower red dashed lines indicated a positive and negative association with *P*-value <0.05, respectively. **(B)** The association of clusters 1, 2, 3, and 4 with age, stage, location (primary or metastatic), and a family history of cancer was shown. Cluster 2 and cluster 4 were found negatively associated with a family history of cancer and age, respectively.

The transformed *P*-value was calculated after Fisher’s exact test between the status of clinical characteristics and each group of patients (**Figure 5A**). Group 1 was significantly associated with PR status; groups 2 and 3 were significantly associated with ER status. Fisher’s exact test was also used to compare the gene cluster and FH status, which indicated that cluster 2 and cluster 4 were found negatively associated with a family history of cancer and age, respectively (**Figure 5B**). The association of breast cancer subtypes and TNM staging with mutation clusters is shown in **Supplementary Figures 3A, B**. No significant associations were found between them. We also analyzed the association between gene mutations and clinical characteristics in the MSK cohort. Cluster 2 showed a significant association with PR status.

### Actionability of Mutational Gene Clusters

**Figure 6:**
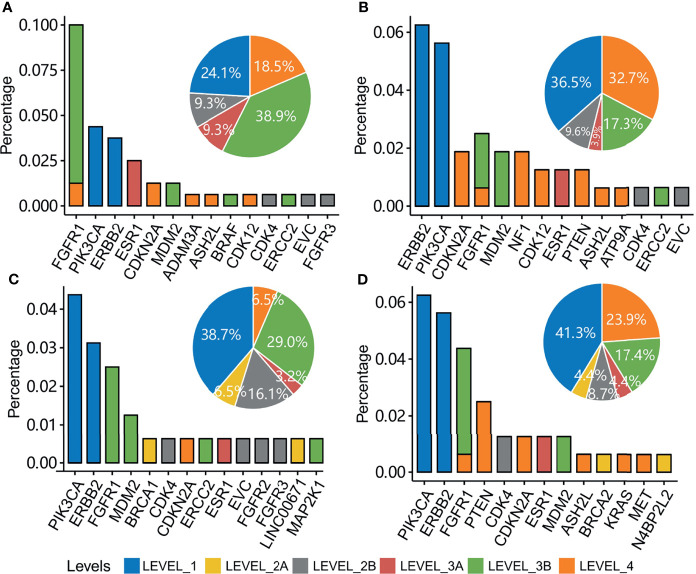
Clinical actionability of somatic alterations for patients with mutations in each patient group. Percentage of patients with actionable genes are shown in patient groups 1 **(A)**, 2 **(B)**, 3 **(C)**, and 4 **(D)**. Bin sizes in the pie plot indicate the related percentage of actionable levels in each patient group.

Mutations in the tumors of the four groups of patients were annotated with the OncoKB database (19). Actionability was classified into six levels according to the confidence in the evidence (**Figure 6**). Level_1 was of the highest evidence, and Level_4 was of the lowest evidence. The highest evidence level, Level_1, was the treatments with an FDA-recognized biomarker predictive of response to an FDA-approved drug, and the lowest evidence level, Level_4, was the treatments with compelling biological evidence that supports the biomarker as being predictive of response to a drug. Patient groups 1, 2, 3, and 4 had 24.1%, 36.5%, 38.7%, and 41.3% of patients with an FDA-recognized biomarker predictive of response to an FDA-approved drug, respectively (**Figure 6**). *ERBB2* and *PIK3CA* were the most enriched actionable genes in groups 2, 3, and 4 with a high evidence level. Although the percentage of actionable mutations in *FGFR1* was high in group 1, they had a low evidence level for majority of the patients.

## Discussion

The role of ER in the treatment of breast cancer can be traced back to Beatson’s work in 1896 (25), which found that ovariectomy could cause regression of metastatic breast cancer. Gradually, antiestrogen therapy became one of the cornerstones of breast cancer treatment. Patients with breast cancer were subtyped according to ER, PR, and HER2 status. With the recent development of genomics, mutations were found to play an important role in cancer development. For example, GATA3 mutation causes ESR1 ligand activation and leads to endocrine therapy resistance (26). However, research had been rare on the role of genomic mutations in the maintenance of ER, PR, and HER2 status. Moreover, familial history could also share some unknown gene mutations. In this study, we made an association study to find the possible candidate mutational genes associated with ER, PR, HER2, and FH status.

We first used gene-by-gene comparison methods to infer the associated genes. Only the top 10 genes were compared between different ER, PR, and HER2 statuses because only a small number of patients had mutated for the other genes. Three significantly mutated genes (*CCND1*, *ERBB2*, and *CDK12*) were found after adjusting multiple-comparison errors with the Benjamin and Hochberg method. MutSigCV did find five significantly mutated genes (*TP53*, *PIK3CA*, *AKT1*, *PTEN*, and *GATA3*). However, neither of them was associated with ER, PR, HER2, and FH status. The statistical power was low because of multiple-comparison errors. For example, discriminating *FGFR1* between the PR+ and PR- groups will need about 900 patients per group. To reduce multiple-comparison times, genes were clustered first. Gene clustering was performed according to gene mutation status across samples. Fisher’s exact test was used to compare each pair of genes. A similarity score was inferred using the resulting *P*-value. Genes were clustered with similarity scores. Four clusters of genes with high within-cluster similarity were identified.

Cluster 1 was significantly associated with PR status and contained five genes (*ADGRA2*, *ZNF703*, *FGFR1*, *KAT6A*, and *POLB*). *ZNF703* was found a common luminal B breast cancer oncogene (27). *FGFR1* amplification was positively associated with luminal B breast cancer (28). In this work, *FGFR1* mutations were either amplification (83.3%) or gene fusion (16.7%). *POLB* mutations were reported to be positively associated with PR expression in gastric cancer (29). However, no report was found about breast cancer. This cluster could be related to the maintenance of PR status.

Cluster 2 was significantly associated with ER status. Cluster 2 had five genes (*COL1A1*, *AXIN2*, *ZNF217*, *GNAS*, and *BRIP1*). A study showed that *COL1A1* was positively associated with ER and PR expression (30). In this work, *COL1A1* mutations were mostly gene amplification (72.7%) and gene fusion (9.0%). In ER+ breast cancer, *ZNF217* worked as a positive enhancer of ER (31). In this work, 85% of *ZNF217* mutations were gene amplification and gene fusion, which could bring about a higher expression of *ZNF217*.

Cluster 3 was positively associated with ER status. Four genes (*FGF3*, *FGF4*, *FGF19*, and *CCND1*) were included in this cluster. The co-amplification of *FGF3*, *FGF4*, *FGF19*, and *CCND1*was also observed in another pan-cancer study from the United States (32). The co-amplification of these genes was caused by large segmental duplication in chromosome 11q13. The coincidence of *CCND1* with ER-positive breast cancer was also observed previously (7, 8).

Cluster 4 was not significantly associated with ER, PR, HER2, or FH status. This cluster consisted of four genes (*PTK2*, *MYC*, *NBN*, and *RAD21*). Most mutations in these genes were amplification. The amplification of *PTK2*, *MYC*, *NBN*, and *RAD21* found prognostic biomarkers independent of breast cancer subtype (33). Such a phenomenon prompted the invalidity of ER, PR, and HER2 on the subtyping of these patients with breast cancer.

Family history could increase the risk of breast cancer. Indeed, the distribution of the cancers diagnosed in the family members of the 160 patients was not random. Lung cancer, liver cancer, breast cancer, endometrial cancer, and colorectal cancer were present in most of their family members. As far as is known, breast cancer, endometrial cancer, and colorectal cancer in any close family member could be risk factors for breast cancer (34–36). A higher percentage of breast cancer, endometrial cancer, and colorectal cancer in family members can be expected. To our surprise, the percentage of liver cancer was significantly higher than that of breast cancer. It may imply that liver cancer in any close family member could also be a risk factor for breast cancer. Lung cancer itself in the population was higher than other cancers. The high proportion of lung cancer in family members of patients with breast cancer could come from either the high background incidence rate or the association with breast cancer, which cannot be discriminated against using our current data.

Considering the high percentage of family history-related cancer, we were curious about the somatic gene mutations associated with FH. The rationale behind this hypothesis is that the similar genomic polymorphism and environment in the family members could cause a subtype of breast cancer associated with FH. However, no positively associated gene mutations or mutational clusters were found. There was only one negatively associated gene cluster (cluster 2). Although the significance of this cluster was still unclear, it shed light on the pathology of these Chinese patients with breast cancer.

The underlying etiology was predicted with mutational signature analysis using an R package “deconstructSigs” (37). The mutational spectrum in the tumors of the four patient groups was compared against COSMIC (Catalogue Of Somatic Mutations In Cancer) signatures (v2.0). For mutations in the tumors, patient groups 1 (**Supplementary Figure 4A**) and 2 (**Supplementary Figure 4B**) were more affected by signature 13 (most common in cervical and bladder cancers, attributed to the activity of the AID/APOBEC family), patient group 3 by signature 1 (endogenous mutational process initiated by spontaneous deamination of 5-methylcytosine) (**Supplementary Figure 4C**), and patient group 4 by signature 6 (defective DNA mismatch repair) (**Supplementary Figure 4D**).

In comparison to the MSK cohort, this cohort showed a different gene mutation spectrum when considering the breast cancer subtypes and ER, PR, and HER2 statuses (**Supplementary Figure 5**). It suggested that there were different mechanisms in the two populations. For example, this cohort had higher proportions of *ERBB2* mutations in the HER2-enriched subtype but the MSK cohort had a similar mutation frequency between the HER2-enriched subtype and the other patients’ clinical characteristics. Such difference would lead to different treatment strategies in different populations.

## Conclusion

Through the association study, we revealed that some genomic mutations and mutational clusters were significantly associated with ER and PR status. The identification of these genomic mutations could help to improve endocrine therapy.

## Data Availability Statement

The original contributions presented in the study are included in the article/**Supplementary Material**. Further inquiries can be directed to the corresponding authors.

## Ethics Statement

The studies involving human participants were reviewed and approved by the Research Ethics Committee of Tianjin Medical University Cancer Institute and Hospital. The patients/participants provided their written informed consent to participate in this study.

## Author Contributions

ZT, YZ, and LL designed the study. CH, CW, NL, WZ, SL, LZ, WM, SW, and YZ performed the data collection and analysis. CH, ZT, YZ, and LL prepared the manuscript. All authors contributed to the article and approved the submitted version.

## Funding

This work is supported by Tianjin Key Medical Discipline (Specialty) Construction Project and Tianjin Medical University Cancer Hospital "14th Five-Year" Peak Discipline Support Program Project. The funders have no role in study design, data collection and analysis, decision to publish, or preparation of the manuscript.

## Conflict of Interest

YZ and LL are employees of Shanghai OrigiMed Co., Ltd.

The remaining authors declare that the research was conducted in the absence of any commercial or financial relationships that could be construed as a potential conflict of interest.

## Publisher’s Note

All claims expressed in this article are solely those of the authors and do not necessarily represent those of their affiliated organizations, or those of the publisher, the editors and the reviewers. Any product that may be evaluated in this article, or claim that may be made by its manufacturer, is not guaranteed or endorsed by the publisher.

## Supplementary Material

Supplementary Figure 1 Cancer distribution in the family members of patients with breast cancer. **(A)** Relative proportion of patients with breast cancer whose family members have any type of cancer is indicated with the bin sizes. **(B)** The percentage of patients with breast cancer whose family members have any type of cancer is sorted along the x-axis.

Supplementary Figure 2 Comparison of gene mutations with MSK cohort. Top mutated genes are compared between the patients with the subtypes of Luminal **(A)**, HER2_enriched **(B)**, and TNBC **(C)** in this study. In the MSK cohort, the difference between ER **(D)**, PR **(E)**, and HER2 **(F)** statuses are also shown. And top mutated genes are compared between the patients with the subtypes of Luminal **(G)**, HER2_enriched **(H)**, and TNBC **(I)**. *, adjusted *P*-value <0.05; **, adjusted *P*-value <0.01; ***, adjusted *P*>-value <0.001.

Supplementary Figure 3 The association between patient groups and breast cancer subtypes (Luminal, HER2, and TNBC). **(A)** The association between gene clusters and breast cancer subtypes is indicated with association scores. Association scores above the upper and below the lower red dashed lines indicate a positive and negative association with *P*-value<0.05, respectively. **(B)** Association between gene clusters and breast cancer TNM staging is indicated with association scores.

Supplementary Figure 4 Deconstruct the signatures in the four groups of patients. The weights of COSMIC (Catalogue Of Somatic Mutations In Cancer) signatures are deconstructed from somatic mutations in the four patient groups using an R package “deconstructSigs”. There are 30 COSMIC signatures (v2.0). The relative importance of signatures is shown with a pie plot for the patient group 1 **(A)**, 2 **(B)**, 3 **(C)**, and 4 **(D)**. A larger area indicates higher importance.

Supplementary Figure 5 The association between patient groups and clinical characteristics in the MSK cohort. The association between gene clusters and clinical characteristics is indicated with association scores in the MSK cohort. Association scores above the upper and below the lower red dashed lines indicate a positive and negative association with *P*-value<0.05, respectively.

Supplementary Table 1 The gene list of sequencing panel.
