# Supplementary figures and images for: Gene Mutations Associated With Clinical Characteristics in the Tumors of Patients With Breast Cancer
Source: Front Oncol. 2022 Apr 14;12:778511. doi: 10.3389/fonc.2022.778511 (PMC9046571; doi:10.3389/fonc.2022.778511)

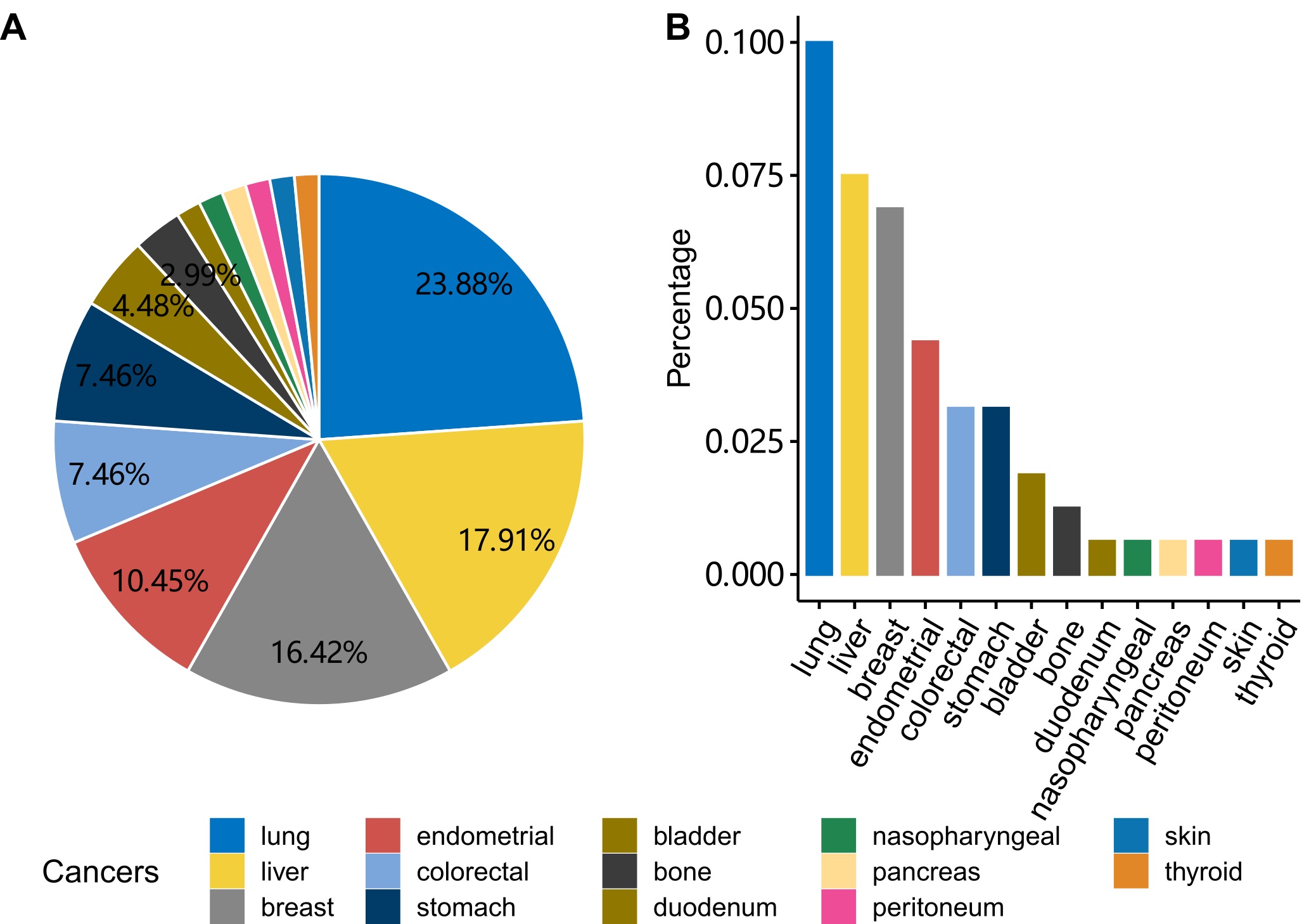

Supplement: Supplementary Figure 1 — Cancer distribution in the family members of patients with breast cancer. (A) Relative proportion of patients with breast cancer whose family members have any type of cancer is indicated with the bin sizes. (B) The percentage of patients with breast cancer whose family members have any type of cancer is sorted along the x-axis. [file Image_1.jpeg]

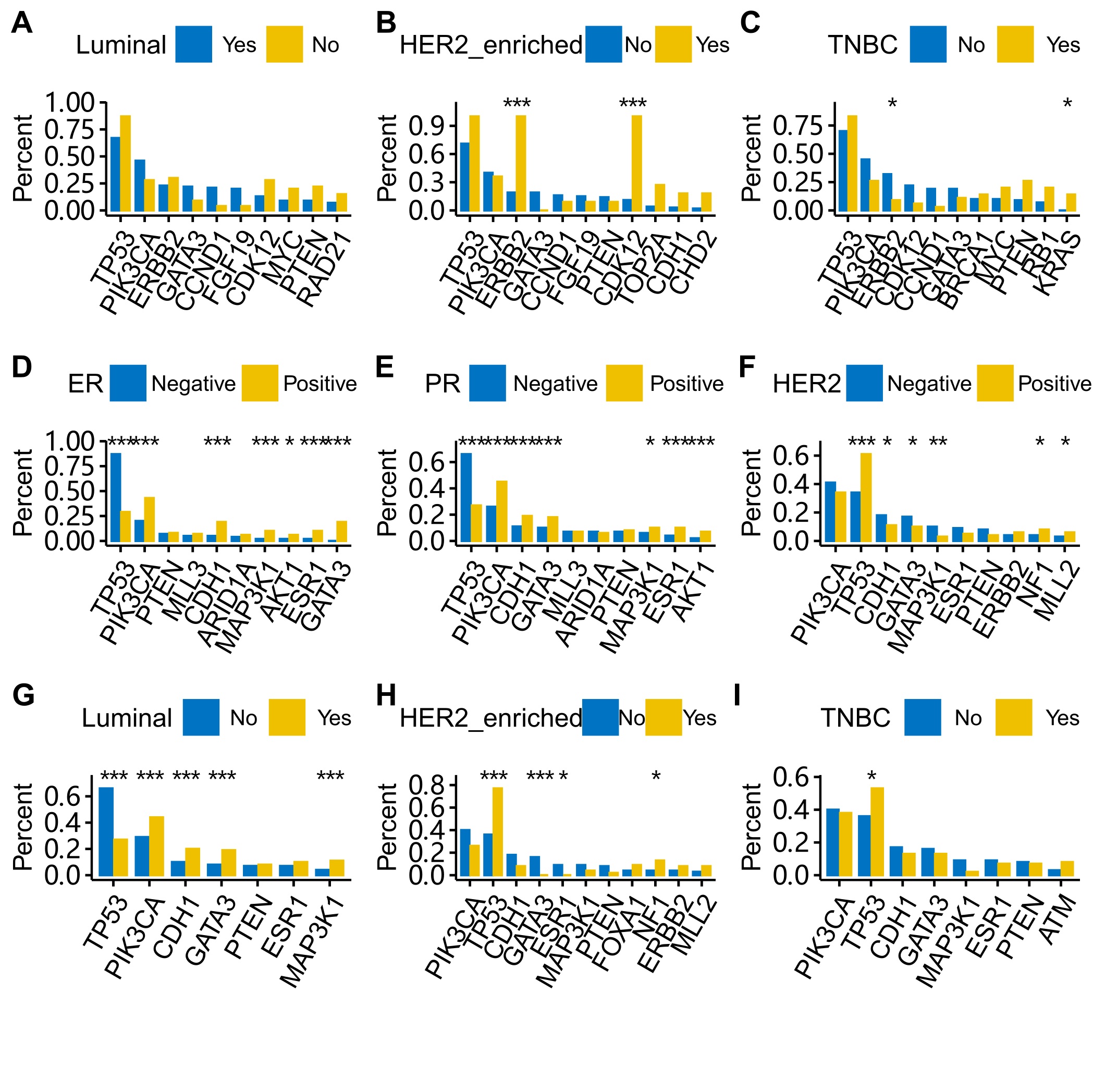

Supplement: Supplementary Figure 2 — Comparison of gene mutations with MSK cohort. Top mutated genes are compared between the patients with the subtypes of Luminal (A), HER2_enriched (B), and TNBC (C) in this study. In the MSK cohort, the difference between ER (D), PR (E), and HER2 (F) statuses are also shown. And top mutated genes are compared between the patients with the subtypes of Luminal (G), HER2_enriched (H), and TNBC (I). *, adjusted P-value <0.05; **, adjusted P-value <0.01; ***, adjusted P>-value <0.001. [file Image_2.jpg]

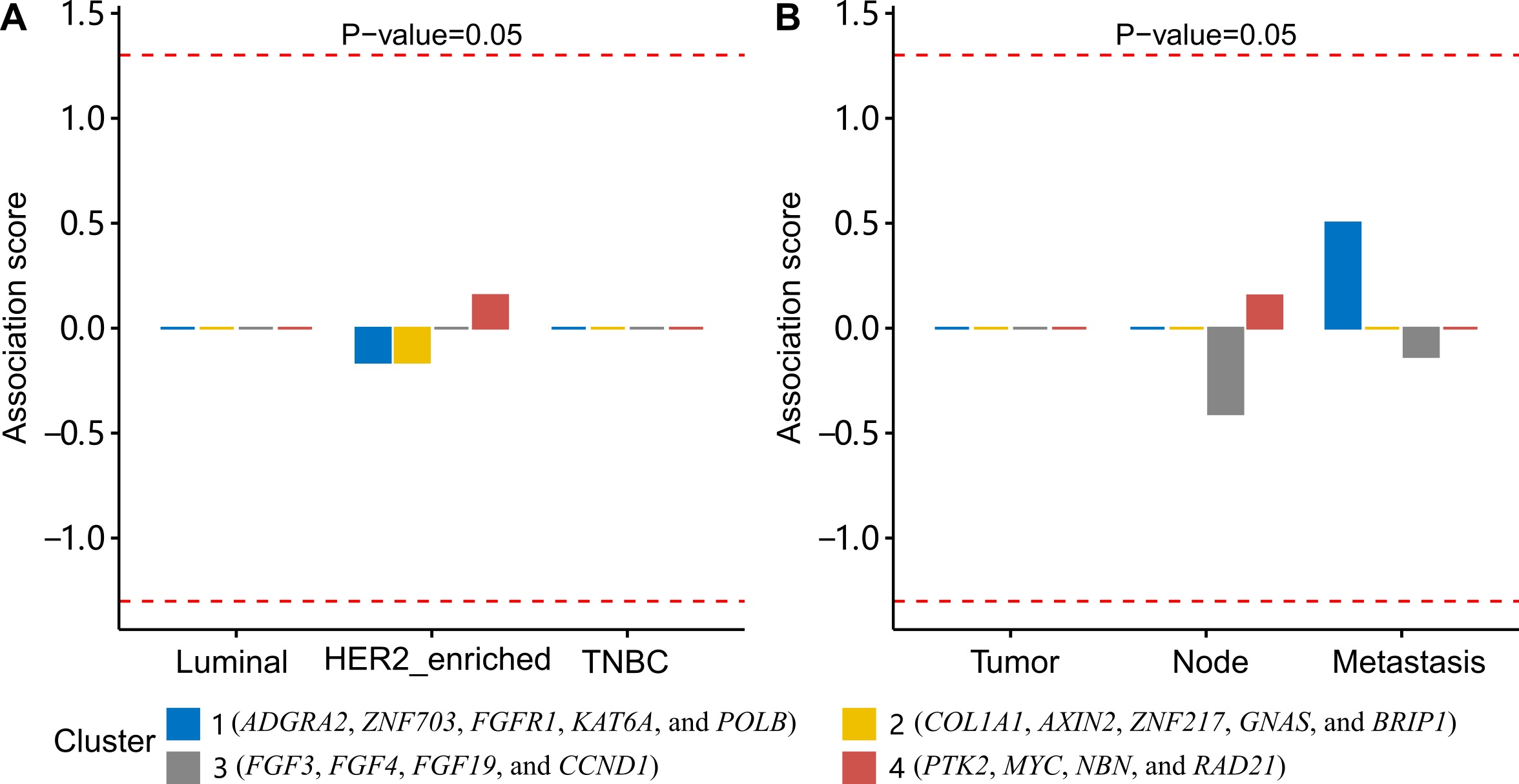

Supplement: Supplementary Figure 3 — The association between patient groups and breast cancer subtypes (Luminal, HER2, and TNBC). (A) The association between gene clusters and breast cancer subtypes is indicated with association scores. Association scores above the upper and below the lower red dashed lines indicate a positive and negative association with P-value<0.05, respectively. (B) Association between gene clusters and breast cancer TNM staging is indicated with association scores. [file Image_3.jpeg]

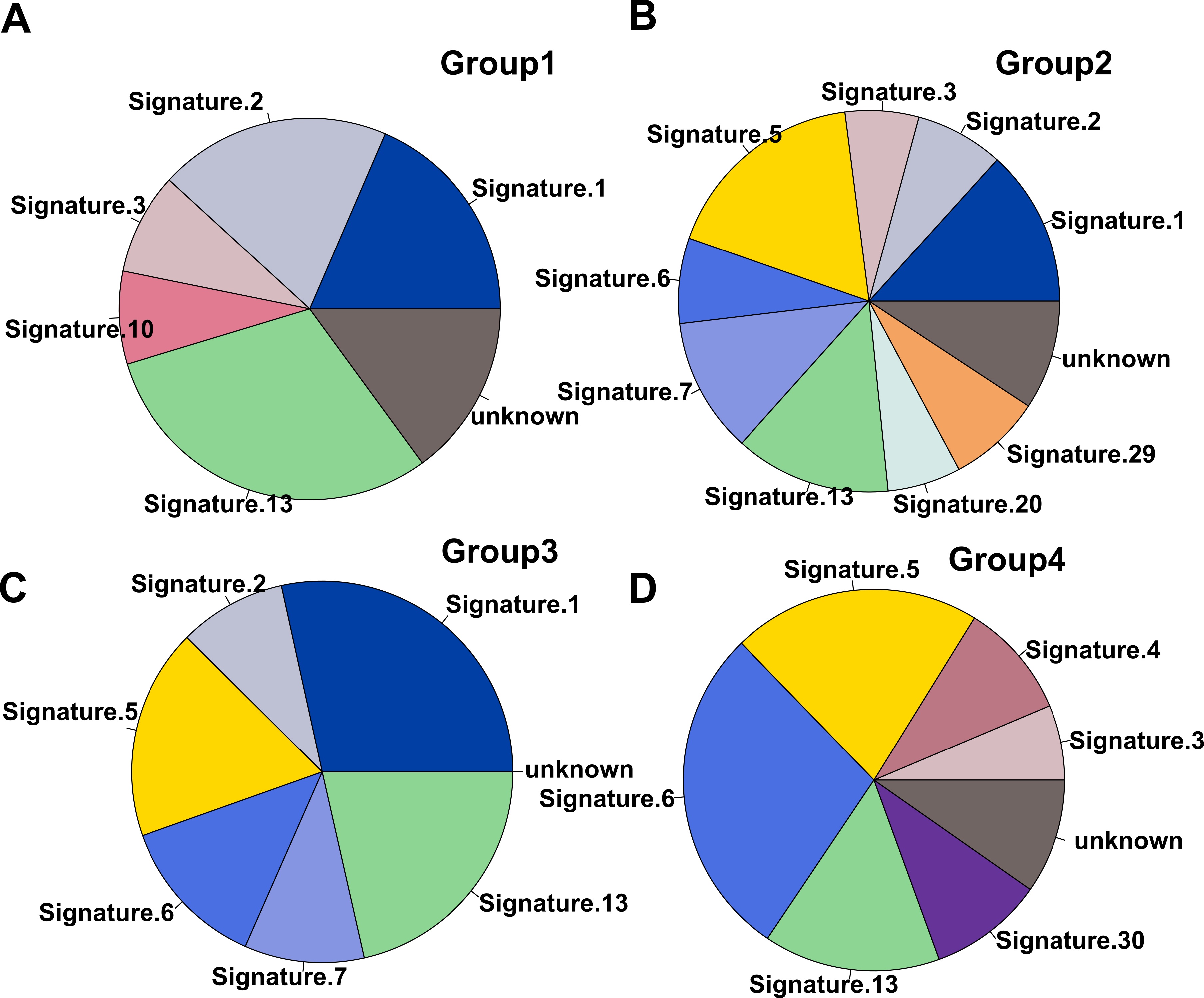

Supplement: Supplementary Figure 4 — Deconstruct the signatures in the four groups of patients. The weights of COSMIC (Catalogue Of Somatic Mutations In Cancer) signatures are deconstructed from somatic mutations in the four patient groups using an R package “deconstructSigs”. There are 30 COSMIC signatures (v2.0). The relative importance of signatures is shown with a pie plot for the patient group 1 (A), 2 (B), 3 (C), and 4 (D). A larger area indicates higher importance. [file Image_4.jpg]

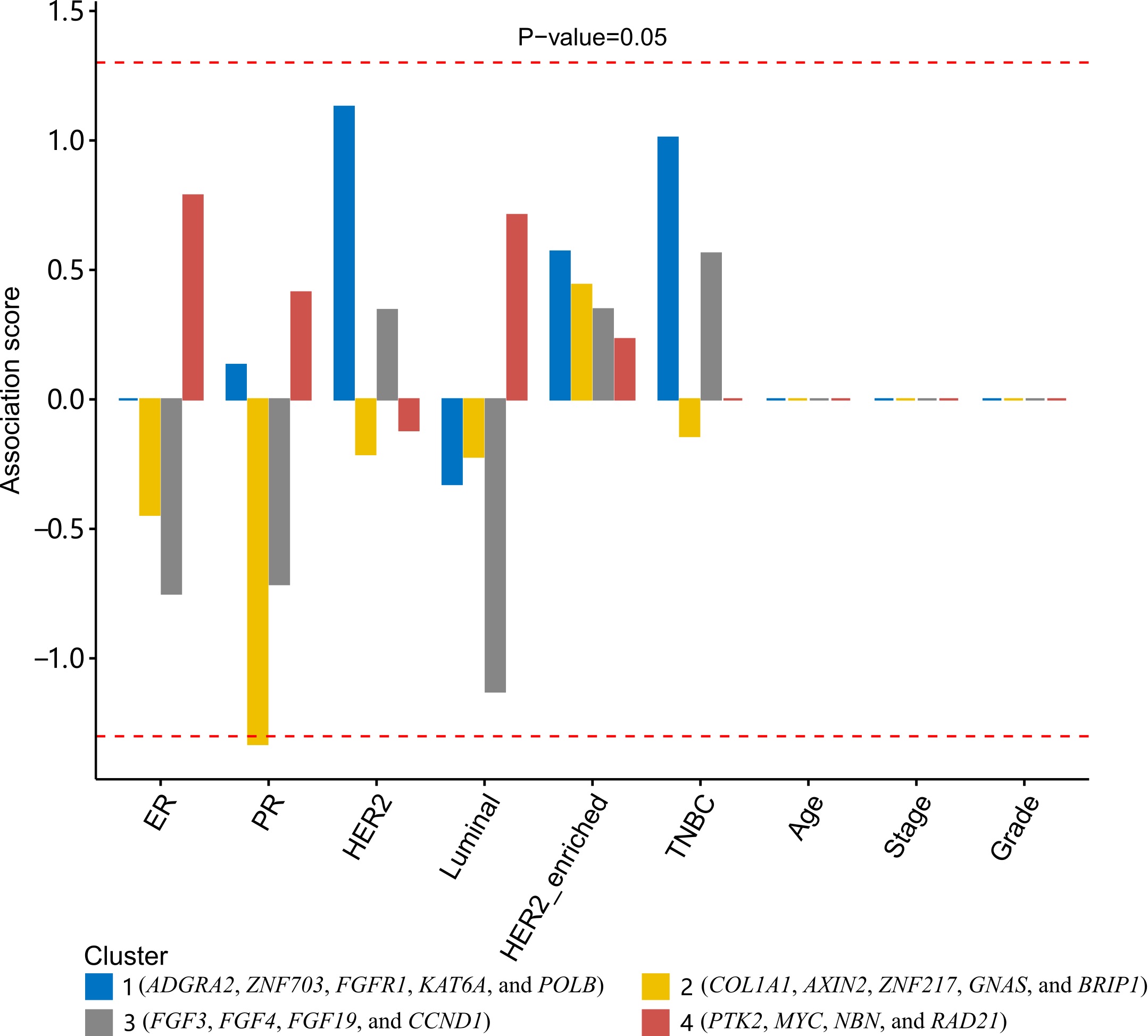

Supplement: Supplementary Figure 5 — The association between patient groups and clinical characteristics in the MSK cohort. The association between gene clusters and clinical characteristics is indicated with association scores in the MSK cohort. Association scores above the upper and below the lower red dashed lines indicate a positive and negative association with P-value<0.05, respectively. [file Image_5.jpeg]
