# Supplementary material for: Gene Mutations Associated With Clinical Characteristics in the Tumors of Patients With Breast Cancer
Source: Front Oncol. 2022 Apr 14;12:778511. doi: 10.3389/fonc.2022.778511 (PMC9046571; doi:10.3389/fonc.2022.778511)
Supplement: Supplementary Table 1 — The gene list of sequencing panel. [file Table_1.docx]

| ABCB1 | ABL1 | ABL2 | ABRAXAS1 | ACVR1 | ACVR1B | ACVR2A | ADAM29 |
| --- | --- | --- | --- | --- | --- | --- | --- |
| ADGRA2 | AFF3 | AKT1 | AKT2 | AKT3 | ALK | AMER1 | ANTXR2 |
| APC | APEX1 | APOBEC3B | AQP3 | AR | ARAF | ARAP3 | ARFRP1 |
| ARHGAP4 | ARHGAP6 | ARHGDIA | ARHGEF10 | ARHGEF25 | ARHGEF3 | ARID1A | ARID1B |
| ARID2 | ASXL1 | ATM | ATR | ATRX | AURKA | AURKB | AXIN1 |
| AXIN2 | AXL | B2M | BAP1 | BARD1 | BCAR4 | BCL2 | BCL2L1 |
| BCL2L11 | BCL2L2 | BCL6 | BCL7A | BCOR | BCORL1 | BCR | BIRC3 |
| BIRC5 | BLK | BLM | BMPR1A | BMX | BRAF | BRCA1 | BRCA2 |
| BRD4 | BRIP1 | BTG1 | BTG2 | BTK | BUB1 | CALR | CAMTA1 |
| CARD11 | CASP8 | CBFB | CBL | CCN6 | CCNB3 | CCND1 | CCND2 |
| CCND3 | CCNE1 | CD1A | CD1B | CD1C | CD1D | CD1E | CD274 |
| CD36 | CD70 | CD74 | CD79A | CD79B | CDC42 | CDC73 | CDH1 |
| CDK12 | CDK2 | CDK4 | CDK6 | CDK8 | CDKN1A | CDKN1B | CDKN2A |
| CDKN2B | CEBPA | CFTR | CHD2 | CHD4 | CHEK1 | CHEK2 | CIC |
| CLDN18 | CNOT2 | COL1A1 | CRBN | CREB3L1 | CREB3L2 | CREBBP | CRKL |
| CRLF2 | CSF1R | CSF3R | CSK | CSNK1A1 | CTCF | CTNNA1 | CTNNA2 |
| CTNNB1 | CUL3 | CUL4A | CXCR4 | CYLD | CYP17A1 | CYP2D6 | DAXX |
| DCTN1 | DDR1 | DDR2 | DEF6 | DEK | DGCR8 | DICER1 | DIS3 |
| DLC1 | DNMT3A | DNMT3B | DOT1L | DPYD | DYNC1H1 | DYNLL1 | E2F3 |
| ECT2 | EED | EGF | EGFR | EMSY | EP300 | EPAS1 | EPCAM |
| EPHA2 | EPHA3 | EPHA5 | EPHA6 | EPHA7 | EPHA8 | EPHB1 | EPHB4 |
| ERBB2 | ERBB3 | ERBB4 | ERCC1 | ERCC2 | ERCC3 | ERCC4 | ERCC5 |
| ERG | ERRFI1 | ESR1 | ETV1 | ETV4 | ETV5 | ETV6 | EWSR1 |
| EZH2 | EZR | FAM135B | FAM46C | FANCA | FANCC | FANCD2 | FANCE |
| FANCF | FANCG | FANCI | FANCL | FANCM | FARP1 | FAS | FAT1 |
| FAT3 | FAT4 | FBXO31 | FBXW7 | FEV | FGF1 | FGF10 | FGF12 |
| FGF14 | FGF18 | FGF19 | FGF2 | FGF21 | FGF23 | FGF3 | FGF4 |
| FGF5 | FGF6 | FGF7 | FGF9 | FGFR1 | FGFR2 | FGFR3 | FGFR4 |
| FGR | FH | FLCN | FLI1 | FLT1 | FLT3 | FLT4 | FNDC3B |
| FOS | FOXA1 | FOXL2 | FOXO1 | FRS2 | FUBP1 | FUS | FYN |
| GABRA6 | GALNT12 | GATA1 | GATA2 | GATA3 | GATA4 | GATA6 | GEN1 |
| GID4 | GLI1 | GLI2 | GLI3 | GNA11 | GNA13 | GNAQ | GNAS |
| GREM1 | GRIN2A | GRM3 | GSK3B | H2AX | H3-3B | H3C2 | HCK |
| HDAC1 | HDAC2 | HDAC6 | HDAC9 | HGF | HMGA1 | HMGA2 | HNF1A |
| HRAS | HSD3B1 | HSP90AA1 | HTATIP2 | ID3 | IDH1 | IDH2 | IDO1 |
| IGF1R | IGF2 | IKBKB | IKBKE | IKZF1 | IL7R | INHBA | INPP4B |
| IRF2 | IRF4 | IRS2 | ITK | JAK1 | JAK2 | JAK3 | JAZF1 |
| JUN | KAT6A | KDM5A | KDM5B | KDM5C | KDM6A | KDR | KEAP1 |
| KEL | KLF5 | KLHL6 | KMT2A | KMT2C | KMT2D | KNSTRN | KRAS |
| LCK | LIMK1 | LMO1 | LRIG1 | LRP1 | LRP1B | LRP2 | LTK |
| LYN | LZTR1 | MACC1 | MAF | MAGI2 | MALAT1 | MAML2 | MAP2K1 |
| MAP2K2 | MAP2K4 | MAP3K1 | MAP3K13 | MAP4K5 | MAPK1 | MAX | MCF2L |
| MCL1 | MDM2 | MDM4 | MECOM | MED12 | MEF2B | MEN1 | MET |
| MGMT | MITF | MKNK1 | MLH1 | MLLT3 | MR1 | MRE11 | MS4A1 |
| MSH2 | MSH3 | MSH6 | MST1R | MTAP | MTG1 | MTOR | MUC16 |
| MUTYH | MYB | MYBL1 | MYC | MYCL | MYCN | MYD88 | MYH11 |
| MYOD1 | NAB2 | NBN | NCOA2 | NCOR1 | NECTIN4 | NEK11 | NET1 |
| NF1 | NF2 | NFE2L2 | NFIB | NFKBIA | NKX2-1 | NOTCH1 | NOTCH2 |
| NOTCH3 | NOTCH4 | NPAT | NPM1 | NR4A3 | NRAS | NRG1 | NRG3 |
| NSD1 | NSD2 | NSD3 | NT5C2 | NTHL1 | NTRK1 | NTRK2 | NTRK3 |
| NUP88 | NUP93 | NUTM1 | OBSCN | P2RY8 | PAK1 | PAK3 | PALB2 |
| PARP1 | PARP2 | PARP3 | PARP4 | PAX3 | PAX5 | PAX7 | PBRM1 |
| PBX1 | PCA3 | PDCD1 | PDCD1LG2 | PDGFB | PDGFRA | PDGFRB | PDK1 |
| PHF6 | PHOX2B | PIK3C2B | PIK3C2G | PIK3C3 | PIK3CA | PIK3CB | PIK3CD |
| PIK3CG | PIK3R1 | PIK3R2 | PIM1 | PKD2 | PKN1 | PLA2G1B | PLCG2 |
| PML | PMS2 | POLB | POLD1 | POLE | POT1 | PPARG | PPP2R1A |
| PRDM1 | PREX2 | PRKACA | PRKACB | PRKAR1A | PRKCI | PRKDC | PRKN |
| PRPF38B | PRSS1 | PRSS8 | PTCH1 | PTEN | PTK2 | PTK6 | PTPN11 |
| PTPRT | QKI | RAC1 | RAD21 | RAD50 | RAD51 | RAD51B | RAD51C |
| RAD51D | RAD52 | RAD54B | RAD54L | RAF1 | RANBP2 | RARA | RASA1 |
| RB1 | RBBP8 | RBM10 | RECQL | RECQL4 | REL | RELA | RELB |
| RET | REV3L | RGS7 | RHBDF2 | RHEB | RHOA | RICTOR | RIT1 |
| RNASEL | RNF43 | ROCK2 | ROS1 | RPTOR | RSPO2 | RSPO3 | RUNX1 |
| RUNX1T1 | RXRA | SDC4 | SDHA | SDHAF2 | SDHB | SDHC | SDHD |
| SERPINB3 | SERPINB4 | SETBP1 | SETD2 | SF3B1 | SGK1 | SHQ1 | SIK1 |
| SKP2 | SLC1A2 | SLC34A2 | SLC6A2 | SLIT2 | SLX4 | SMAD2 | SMAD3 |
| SMAD4 | SMARCA2 | SMARCA4 | SMARCD1 | SMARCE1 | SMO | SNCAIP | SND1 |
| SOCS1 | SOX2 | SOX9 | SPEN | SPINK1 | SPOP | SPTA1 | SRC |
| SRGAP1 | SRMS | SRSF2 | SS18 | SSX1 | STAG2 | STAT3 | STAT4 |
| STAT6 | STK11 | STK24 | SUFU | SUZ12 | SYK | TACSTD2 | TAF1 |
| TARBP2 | TBX3 | TCF3 | TCF7L2 | TEK | TENT5C | TERC | TERT |
| TET1 | TET2 | TET3 | TFE3 | TFEB | TGFBR1 | TGFBR2 | TIE1 |
| TIPARP | TMEM127 | TMPRSS2 | TNFAIP3 | TNFRSF14 | TNFRSF19 | TNFSF11 | TNFSF13B |
| TNK2 | TOP1 | TOP2A | TP53 | TP63 | TPMT | TRAF7 | TRIO |
| TSC1 | TSC2 | TSHR | TSPAN1 | TSPAN31 | TYK2 | TYRO3 | U2AF1 |
| UGT1A1 | USP6 | VEGFA | VGLL3 | VHL | WEE1 | WEE2 | WISP3 |
| WNK1 | WRN | WT1 | XPO1 | XRCC2 | XRCC3 | YAP1 | YES1 |
| YWHAE | ZBTB2 | ZFHX3 | ZNF217 | ZNF703 | ZNF750 | ZNRF3 | ZRSR2 |
