# Supplementary material for: Gene Mutations Associated With Clinical Characteristics in the Tumors of Patients With Breast Cancer
Source: Front Oncol. 2022 Apr 14;12:778511. doi: 10.3389/fonc.2022.778511 (PMC9046571; doi:10.3389/fonc.2022.778511)
Supplement: Supplementary file 7 [file Table_2.docx]

Supplemental Table 2. **Gene mutations in breast cancer and corresponding drugs with evidence**

| Gene | Levels | Drug | Annotation | Type |
| --- | --- | --- | --- | --- |
| PTEN | LEVEL_4 | AZD8186,GSK2636771 | Missense_Mutation | SNV |
| PIK3CA | LEVEL_1 | Fulvestrant+Alpelisib | Missense_Mutation | SNV |
| CDKN2A | LEVEL_4 | Abemaciclib,Palbociclib,Ribociclib | Missense_Mutation | SNV |
| ESR1 | LEVEL_3A | AZD9496,Fulvestrant | Missense_Mutation | SNV |
| KRAS | LEVEL_4 | Binimetinib,Cobimetinib,Trametinib | Missense_Mutation | SNV |
| NF1 | LEVEL_4 | Cobimetinib,Trametinib | Nonsense_Mutation | SNV |
| MAP2K1 | LEVEL_3B | Cobimetinib,Trametinib | Missense_Mutation | SNV |
| CDKN2A | LEVEL_4 | Abemaciclib,Palbociclib,Ribociclib | Nonsense_Mutation | SNV |
| ERBB2 | LEVEL_3A | Neratinib | Missense_Mutation | SNV |
| ERCC2 | LEVEL_3B | Cisplatin | Nonsense_Mutation | SNV |
| NF1 | LEVEL_4 | Cobimetinib,Trametinib | Missense_Mutation | SNV |
| PIK3CA | LEVEL_1 | Fulvestrant+Alpelisib | 3'Flank | SNV |
| BRCA2 | LEVEL_2A | Olaparib,Talazoparib | Nonsense_Mutation | SNV |
| BRCA1 | LEVEL_2A | Olaparib,Talazoparib | Deletion | CNV |
| CDK4 | LEVEL_2B | Abemaciclib,Palbociclib | Amplification | CNV |
| CDKN2A | LEVEL_4 | Abemaciclib,Palbociclib,Ribociclib | Deletion | CNV |
| ERBB2 | LEVEL_1 | Ado-Trastuzumab Emtansine,Lapatinib,  Neratinib,Pertuzumab+Trastuzumab,  Trastuzumab,Trastuzumab+Lapatinib | Amplification | CNV |
| FGFR1 | LEVEL_3B | AZD4547,BGJ398,Debio1347,Erdafitinib | Amplification | CNV |
| MDM2 | LEVEL_3B | Milademetan Tosylate,RO5045337 | Amplification | CNV |
| NF1 | LEVEL_4 | Cobimetinib,Trametinib | Deletion | CNV |
| PTEN | LEVEL_4 | AZD8186,GSK2636771 | Deletion | CNV |
| LINC00671 | LEVEL_2A | Olaparib,Talazoparib | BRCA1 | Fusion |
| BRCA2 | LEVEL_2A | Olaparib,Talazoparib | N4BP2L2 | Fusion |
| BRCA2 | LEVEL_2A | Olaparib,Talazoparib | KCNH5 | Fusion |
| FGFR1 | LEVEL_4 | AZD4547,BGJ398,Debio1347,Erdafitinib | ASH2L | Fusion |
| KIF5B | LEVEL_2B | Cabozantinib,Vandetanib | RET | Fusion |
| ETV6 | LEVEL_1 | Larotrectinib | NTRK3 | Fusion |
| BRAF | LEVEL_3B | Cobimetinib,Trametinib | intergenic | Fusion |
| BRAF | LEVEL_3B | Cobimetinib,Trametinib | NRCAM | Fusion |
| SPACA6 | LEVEL_3B | Cisplatin | ERCC2 | Fusion |
| FGFR3 | LEVEL_2B | Erdafitinib | EVC | Fusion |
| NTRK3 | LEVEL_1 | Larotrectinib | BORCS5 | Fusion |
| ESR1 | LEVEL_3A | AZD9496,Fulvestrant | SYNE1 | Fusion |
| TSC2 | LEVEL_2B | Everolimus | intergenic | Fusion |
| CDK12 | LEVEL_4 | Cemiplimab,Nivolumab,Pembrolizumab | LINC01982 | Fusion |
| MET | LEVEL_4 | Crizotinib | intergenic | Fusion |
| FGFR2 | LEVEL_2B | Erdafitinib | intergenic | Fusion |
| FGFR1 | LEVEL_4 | AZD4547,BGJ398,Debio1347,Erdafitinib | ADAM3A | Fusion |
| ATP9A | LEVEL_4 | Cobimetinib,Trametinib | NF1 | Fusion |
| ZEB1 | LEVEL_4 | Cemiplimab,Nivolumab,Pembrolizumab | CDK12 | Fusion |
| PDGFRA | LEVEL_2B | Imatinib | Intergenic | Fusion |
| FGFR3 | LEVEL_2B | Erdafitinib | RBM47 | Fusion |
| CDK12 | LEVEL_4 | Cemiplimab,Nivolumab,Pembrolizumab | TP53 | Fusion |
| RALYL | LEVEL_2B | Everolimus | TSC2 | Fusion |
